# Tumbling-Snake Model for Polymeric Liquids Subjected to Biaxial Elongational Flows with a Focus on Planar Elongation

**DOI:** 10.3390/polym10030329

**Published:** 2018-03-16

**Authors:** Pavlos S. Stephanou, Martin Kröger

**Affiliations:** 1 Department of Mathematics and Statistics, University of Cyprus, P.O. Box 20537, Nicosia 1678, Cyprus; 2 Polymer Physics, Department of Materials, ETH Zurich, CH-8093 Zurich, Switzerland

**Keywords:** polymer melt, stochastic differential equation, link tension coefficient, entanglements, biaxial flow

## Abstract

We have recently solved the tumbling-snake model for concentrated polymer solutions and entangled melts in the presence of both steady-state and transient shear and uniaxial elongational flows, supplemented by a variable link tension coefficient. Here, we provide the transient and stationary solutions of the tumbling-snake model under biaxial elongation both analytically, for small and large elongation rates, and via Brownian dynamics simulations, for the case of planar elongational flow over a wide range of rates, times, and the model parameters. We show that both the steady-state and transient first planar viscosity predictions are similar to their uniaxial counterparts, in accord with recent experimental data. The second planar viscosity seems to behave in all aspects similarly to the shear viscosity, if shear rate is replaced by elongation rate.

## 1. Introduction

Understanding the behavior of polymer liquids in shearfree (extensional) flows has attracted the interest of academic researchers and industrial companies alike, due to the capacity of such flows to align and stretch polymer chains at a preferred flow direction, such as in fiber spinning and film forming processes [1]. The reliable measurement of uniaxial extensional viscosity has been resolved more than two decades ago with the development of the filament stretching rheometer [2]. Today, this rheometer has reached a level of maturity that allows to demonstrate that systems with the same number of entanglements, and thus with identical linear rheology, have a drastically different nonlinear uniaxial extensional behavior [3,4,5].

On the other hand, the measurement of the planar or biaxial extensional viscosities is rather scarce and mainly unable to reach the steady-state (see e.g., [6,7]), while the such flow fields can be generated and controlled conveniently via optical birefringence in a cross-slot channel [8,9,10]. Rheooptics is then applied to interpret the data. The unavailability of reliable direct rheological data for planar elongation may be the reason for only a few works [11,12,13,14] devoted to testing the ability of rheological constitutive models to address this flow. Non-equilibrium molecular dynamics (NEMD) simulation of microscopic polymer chain models has helped in the past to clarify the applicability of constitutive relationships for simple flows, including uniaxial elongational and shear flows, while it is worthwhile recalling that steady-state planar elongation is easier to implement than uniaxial elongation (UE) in such a simulation setup [15,16,17].

Since the introduction of the tube/reptation concept by de Gennes and Doi & Edwards [18,19,20], this mean-field theory turned out to serve as the far-most capable starting point in an attempt to describe the dynamical nonequilibrium behavior of entangled (high molecular weight) polymer melts and concentrated polymer solutions. At equilibrium, the incorporation of additional mechanisms, such as contour length fluctuations and constraint release (CR) [19,21,22], lead to an accurate description of linear viscoelastic properties [21,22,23,24]; under flow, however, and despite numerous modifications such as the consideration of chain stretch [25], finite extensibility [26,27], and convective constraint release [27,28,29,30]), it still lacks consistency with available rheological data.

Another formalism that aims to address the rheological response of high molecular weight polymeric melts and concentrated solutions is the model developed by Curtiss & Bird [31,32] based on the phase-space formulation within the kinetic theory of undiluted polymers [33]. It invokes neither a mean field tube nor slip-links. It is also known as the tumbling-snake model [34], as it allows for both orientational and curvilinear diffusion of polymer segments. The model entails, as the original tube/reptation model, the solution of a Fokker-Planck (FP) for the single-link distribution function, f(σ,u,t), which describes the probability that at time *t* a chain segment at contour position σ∈[0,1] along the chain is oriented in direction u, with u and σ independent dynamical variables, and u·u=1. Segmental motion is not considered as a strict one-dimensional diffusion process (“reptation”) along the polymer’s backbone but the chain is also allowed to explore the surrounding space by moving perpendicular to its backbone (that may be identified as CR events) with the parameter $\varepsilon^{\prime }$ controlling its significance. The strictly one-dimensional diffusion process of Doi & Edwards is recovered as a special case, when $\varepsilon^{\prime }=0$. The extra stress tensor, see Equation (1) below, contains a term due to the anisotropy of the friction tensor $\zeta =\zeta_{\mathrm{eq}}\left[\delta -(1-\varepsilon )uu\right]$ involving a link tension coefficient ε∈[0,1]; if ε=0 there is no friction against motion in the direction u, whereas for ε=1 the friction tensor is isotropic as for an individual sphere. Despite the qualitatively different assumptions made by the two formalisms, the original tube/reptation model is obtained as a special case of the more general FP equation of the tumbling-snake model [31,32,33,35] when $\varepsilon^{\prime }=\varepsilon =0$. Only the analytically tractable model with $\varepsilon^{\prime }=0$ had been solved rigorously [31,32,33,36,37].

We have shown recently that the tumbling-snake model for $\varepsilon^{\prime }>0$ can be analyzed conveniently via Brownian Dynamics simulations and applied this approach to both steady-state [34,35,38] and time-dependent shear flow [34,38], as well as to steady-state and time-dependent uniaxial elongation [39]. These works provided evidence that the tumbling-snake model is able to capture the damping behavior of the transient viscosity in start-up shear experiments at high rates [40,41,42], while preserving the absence of such undershoots in both normal stress coefficients, in line with experimental data [34,38]. The appearance of the undershoot has been associated with the shear-induced rotational motion of chains [38,42], further supported by non-equilibrium atomistic simulations [43,44,45]. As such, similar undershoots are not seen in elongational flows [39].

The qualitatively relevant and only modification to the original tumbling-snake model was the consideration of a variable link tension coefficient, that vanishes in the absence of flow, and is given by $\varepsilon =\varepsilon_{0}S_{2}^{2}$ [34,38], where $S_{2}$ denotes the 2nd rank uniaxial nematic order parameter of polymer segments [46]. This adjustment of the Curtiss & Bird theory has eliminated certain disadvantages of their original model (exhibiting a constant link tension coefficient). Due to the refinement, the transient shear and elongational viscosities no longer approach constant values at small times, and spurious time oscillations of the transient second normal stress in startup of shear flow are absent. It has been demonstrated that the tumbling-snake model in its present form is able to qualitatively capture recent experimental evidence according to which the extensional viscosity of polymer solutions is seen to exhibit thinning below the inverse Rouse time and thickening above, whereas the extensional viscosity of polymer melts is monotonically decreasing for all strain rates [3,4,5], by having the strength $\varepsilon_{0}$ of the link tension coefficient increasing as the polymer concentration decreases [39].

In this work, we discuss the solution of the tumbling-snake for the more general case of biaxial elongational flows, with a focus on planar elongational flow. The structure of this manuscript is as follows: In Section 2 we revisit the stress tensor of the tumbling-snake model, parameterize the velocity gradient tensor and define the viscosities. Section 3 summarizes the Brownian dynamics method to solve the model. In Section 4, we provide the series expansion of the two biaxial elongational viscosities in the case of steady-state general shearfree flow for small dimensionless elongation rates for comparison with limiting results presented in Section 6. Similarly, in Section 5 we derive analytic expressions for the linear viscoelastic viscosities in the case of start-up flow, again for biaxial flows. In both sections, we discuss the cases of constant and variable link tension coefficient separately. This includes special cases such as the rigid dumbbell. In Section 6 we actually solve the model numerically using Brownian dynamics simulation for planar elongation, validate the analytic expressions, and further compare the predictions of the first planar viscosity with the uniaxial elongation viscosity. We conclude with Section 7 where we discuss the significance of this work.

## 2. Stress Tensor

**Figure 1:**
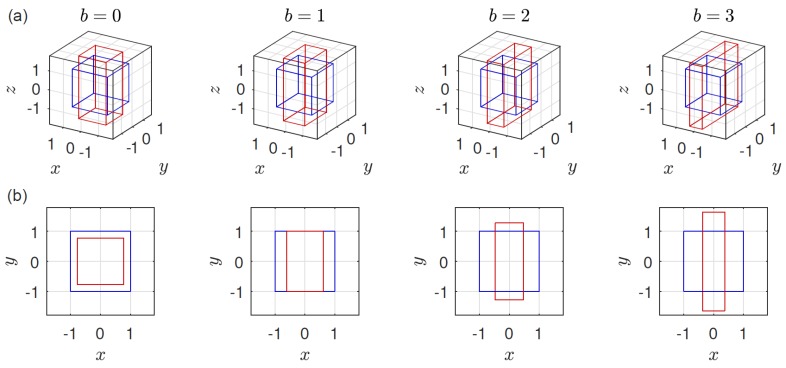
Prominent, qualitatively different examples for isochoric deformations $r^{\prime }$ (red) of a unit cell r (blue) in shearfree flows: (i) uniaxial extension (b=0), (ii) planar extension (b=1), (iii) elliptical extension (b=2), and (iv) equibiaxial extension (b=3). For each scenario the situation is depicted both in (**a**) 3D and (**b**) projected to the *x*–*y*–plane. Coordinates are related via $r^{\prime }=E\cdot r$ by the deformation gradient tensor E=exp(κt), c.f. Equation (3), here shown for $\dot{\epsilon }t=0.5$.

In the case of a monodisperse polymer with “polymerization degree” *N* related to the number of entanglements per chain, *Z*, introduced by Doi and Edwards, via N=3Z, and polymer number density *n*, the time-dependent (extra or polymeric) stress tensor τ of the tumbling-snake model subjected to a homogeneous flow field characterized by the transposed velocity gradient tensor κ is given by [33,35]

(1) $$\begin{array}{ccc} \frac{\tau (t)}{G} & = & -\left(1-\varepsilon^{\prime }\right)\left({\langle \mathrm{uu}\rangle }^{\left(1\right)}\left(t\right)-\frac{1}{3}I\right)-3\varepsilon_{0}^{\prime }\left({\langle \mathrm{uu}\rangle }^{\left(2\right)}\left(t\right)-\frac{1}{18}I\right)-\varepsilon \;B\left(t\right), \end{array}$$

with modulus $G=nk_{B}T\left(N-1\right)$, temperature *T*, Boltzmann’s constant $k_{B}$, unit tensor I, coefficients $\varepsilon^{\prime }$ and $\varepsilon_{0}^{\prime }$ interrelated via $\varepsilon_{0}^{\prime }\equiv \varepsilon^{\prime }{\left(N-1\right)}^{2}$, and a link tension coefficient ε. The latter comes in two versions, the original one proposed by Curtiss & Bird, where ε is a constant, and the variable one proposed by us within the tumbling-snake model, $\varepsilon =\varepsilon_{0}S_{2}^{2}$ with constant $\varepsilon_{0}$ and uniaxial order parameter $S_{2}$ determined by ${\langle \mathrm{uu}\rangle }^{\left(1\right)}\left(t\right)$. Note that we are adopting throughout the nomenclature of Bird et al. [33], while the τ in (1) is a pressure tensor, and thus the negative stress tensor, in the majority of scientific literature. This stress tensor (1) involves the following orientational averages calculated with the solution of the corresponding FP equation [34] for the single-link orientational distribution function f(σ,u,t)

(2) $$\begin{array}{ccc} {\langle \mathrm{uu}\rangle }^{\left(1\right)}\left(t\right) & = & \int_{0}^{1}\;\;d\sigma \int \;du\;f\left(\sigma ,u,t\right)uu\\ {\langle \mathrm{uu}\rangle }^{\left(2\right)}\left(t\right) & = & \int_{0}^{1}\;\;\sigma \left(1-\sigma \right)d\sigma \int \;du\;f\left(\sigma ,u,t\right)uu,\\ B(t) & = & \lambda \kappa :\int_{0}^{1}\;\;\sigma \left(1-\sigma \right)d\sigma \int \;du\;f\left(\sigma ,u,t\right)uuuu, \end{array}$$

where ∫du denotes an integral over the unit sphere, λ a time constant proportional to $\zeta_{\mathrm{eq}}/k_{B}T$, squared bond length *a*, and $N^{3+\beta }$, where β is a chain constraint exponent, and κ:uu=(κ·u)·u stands for a two-fold contraction. The reptation or disengagement time is $\tau_{d}\equiv \lambda /\pi^{2}$. In addition to the highlighted dependency on time *t* the stress tensor as well as the averages depend also on the flow field κ via *f*. For the case of a general shearfree elongational, homogeneous incompressible flow at rate $\dot{\epsilon }$ the transposed velocity gradient tensor κ is traceless and diagonal and thus of the form

(3) $$\kappa =\dot{\epsilon }\left(\begin{array}{ccc} -\frac{1}{2}\left(1+b\right) & 0 & 0\\ 0 & -\frac{1}{2}\left(1-b\right) & 0\\ 0 & 0 & 1 \end{array}\right),\;b\geq 0$$

where it is sufficient to consider semipositive *b* for symmetry reasons. When b=0, we retrieve homogeneous simple uniaxial elongation for $\dot{\epsilon }>0$ and biaxial stretching for $\dot{\epsilon }<0$, while b=1 corresponds to planar elongation [33], b=3 to equibiaxial extension, and b=2 to so-called elliptical extension (Figure 1). Arbitrary *b* can be realized experimentally via a multiaxial elongational rheometer [6]. While most results and methods to be presented below are valid for arbitrary *b*, we will focus on planar elongation (b=1) in Section 6. The ratio of principle strain rates $\kappa_{22}/\kappa_{33}$, denoted by *m* by Demarmels and Meissner [6], is related to *b* by m=(b−1)/2, and the projection of the motion of a material particle in the xz– or yz–plane is given by $x=Cz^{1-m}$ and $y=Cz^{m}$ with constants *C*, while $y\left(t\right)=x\left(t\right)e^{bt}$ in the xy–plane. Except for b=0 ($\kappa_{xx}=\kappa_{yy}$) and b=3 ($\kappa_{yy}=\kappa_{zz}$) there are two normal stress coefficients and corresponding viscosities that can be measured. The first, $\eta_{1}\left(\dot{\epsilon }\right)=-\left(\tau_{zz}-\tau_{xx}\right)/\dot{\epsilon }$, and the second, $\eta_{2}\left(\dot{\epsilon }\right)=-\left(\tau_{yy}-\tau_{xx}\right)/\dot{\epsilon }$, rate-dependent stationary elongational viscosity [33]. The corresponding transient viscosities we denote by $\eta_{1}^{+}\left(\dot{\epsilon },t\right)$ and $\eta_{2}^{+}\left(\dot{\epsilon },t\right)$. The transient viscosities in the linear viscoelastic regime do not depend on rate, are thus denoted by $\eta_{1,2}^{+}\left(t\right)=\lim_{\dot{\epsilon }\to 0}\eta_{1,2}^{+}\left(\dot{\epsilon },t\right)$. Because the elongation rate enters the stress tensor in the combination $\lambda \dot{\epsilon }$, we introduce the dimensionless Weissenberg number

(4) $$\mathrm{Wi}=\dot{\epsilon }\lambda .$$

## 3. Brownian Dynamics Simulation

**Figure 2:**
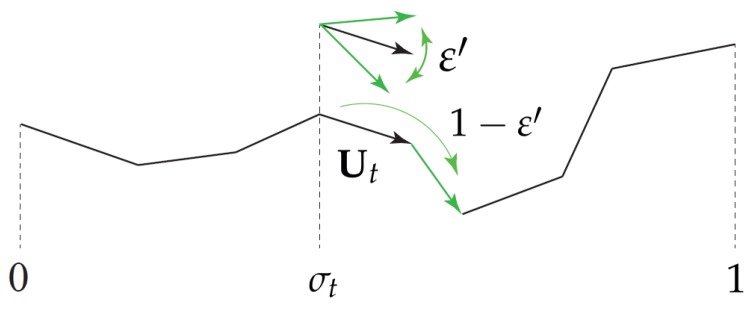
Tumbling-snake model. Strength of the orientational and one-dimensional diffusion of segment vector $U_{t}$ at relative position $\sigma_{t}$ along the polymer contour determined by $\varepsilon^{\prime }\in \left[0,1\right]$. The link-tension coefficient ϵ affects the stress tensor.

The Brownian dynamics algorithm that we employ in this work is identical, apart from the different choice of flow field, κ, with the one we had presented previously. The Fokker-Planck equation of the tumbling-snake model subject to isotropic boundary conditions at chain ends at all times, $\forall_{t}f\left(0,u,t\right)=f\left(1,u,t\right)=1/4\pi$, can be cast in the form of two coupled Itô stochastic differential equations for variables $U_{t}$ (segment unit vector at time *t*) and $\sigma_{t}\in \left[0,1\right]$ (relative contour position at time *t*) as follows

(5) $$\begin{array}{ccc} dU_{t} & = & \left(I\;-\;U_{t}U_{t}\right)\;\cdot \;\left(\kappa \;\cdot \;U_{t}dt+\sqrt{\frac{2\varepsilon_{0}^{\prime }}{\lambda }}dW_{t}\right)\;-\;\frac{2\varepsilon_{0}^{\prime }}{\lambda }U_{t}dt,\\ d\sigma_{t} & = & \sqrt{\frac{2(1-\varepsilon^{\prime })}{\lambda }}dW_{t}^{\prime }, \end{array}$$

where $dW_{t}$ is a three-dimensional Wiener process and $dW_{t}^{\prime }$ is another one-dimensional Wiener process, independent from the former (Figure 2). The transverse projector operator $I-U_{t}U_{t}$ guaranties that the stochastic dynamics preserves the $U_{t}$ property of being a unit vector. Note that the link tension coefficient ε affects the stress tensor, but not the dynamics of the orientational distribution function.

From Equation (5) it is transparent that $1-\varepsilon^{\prime }$ is related to a one-dimensional “reptation” diffusion coefficient, and $\varepsilon_{0}^{\prime }=\varepsilon^{\prime }{\left(N-1\right)}^{2}$ related to the orientational diffusion coefficient of polymer segments. The factor ${\left(N-1\right)}^{2}$ appears because $\sigma_{t}$ is a relative rather than absolute contour position. For implementation details see [34,35].

The moments required to evaluate the stress tensor given by Equation (1), such as ${\langle \mathrm{uu}\rangle }^{\left(1\right)}\left(t\right)$, i.e., the left hand sides of Equation (2), are obtained during Brownian dynamics via plain averaging over an ensemble of stochastic trajectories at times *t*. To be specific, ${\langle \mathrm{uu}\rangle }^{\left(1\right)}\left(t\right)=\langle U_{t}U_{t}\rangle$ and $B\left(t\right)=\lambda \kappa :\langle \sigma_{t}\left(1-\sigma_{t}\right)U_{t}U_{t}U_{t}U_{t}\rangle$ involving the evaluation of a 2nd and 4th rank tensor, whose number of nonvanishing and independent components depends on κ (2 and 6 components, respectively, for the case of biaxial elongation).

## 4. Small Elongation Rate Expansion of the Stationary Viscosities for Biaxial Elongational Flows

Results for the stationary viscosities at small rates can be derived analytically. They are particularly useful because this limiting case can strictly not be accessed during Brownian dynamics, because the error bars increase with decreasing rate. They are furthermore useful to, e.g., test ratios between zero rate viscosities or to compare asymptotic behaviors for different types of flow. The approach to derive analytical results is based on a spherical harmonics expansion of the single-link distribution function around equilibrium.

### 4.1. Stationary Regime, Constant ε

To begin with, we assume a constant ε and we are interested in the rate-dependent steady-state values of the extensional viscosities. The methodology employed is described in detail in the Supplementary Section A and the final expression for the expansion, up to 2nd order in the dimensionless Weissenberg number $\mathrm{Wi}=\dot{\epsilon }\lambda$, is given in Supplementary Equation (S6). Upon inserting this expansion into the stress tensor Equation (1) we obtain both elongational viscosities up to second order in Wi in terms of the biaxiality parameter *b*

(6) $$\begin{array}{ccc} \frac{\eta_{1}\left(\dot{\epsilon }\right)}{G\lambda } & = & \frac{3+b}{60}\left(1\;+\;\frac{2}{3}\varepsilon \right)+\frac{4}{35}\left(1\;-\;\frac{2b}{3}-\;\frac{b^{2}}{3}\right)\left(\;\frac{3}{4}+\varepsilon \right)\Delta_{1}\mathrm{Wi}\\ & & +\frac{12}{245}\left(1\;+\;\frac{b^{2}}{3}\right)\left[\varepsilon \left(\Delta_{2}+6\Delta_{3}\right)\left(1\;+\;\frac{b}{3}\right)+\frac{3}{4}\left(1\;+\;\frac{b^{2}}{3}\right)\left(\Delta_{2}-8\Delta_{3}\right)\right]{\mathrm{Wi}}^{2},\\ \frac{\eta_{2}\left(\dot{\epsilon }\right)}{G\lambda } & = & \frac{b}{30}\left(1\;+\;\frac{2}{3}\varepsilon \right)-\frac{16b}{105}\left(\;\frac{3}{4}+\varepsilon \right)\Delta_{1}\mathrm{Wi}\\ & & +\frac{8b}{245}\left(1\;+\;\frac{b^{2}}{3}\right)\left[\left(\frac{3}{4}+\varepsilon \right)\Delta_{2}+6\left(\varepsilon -1\right)\Delta_{3}\right]{\mathrm{Wi}}^{2}, \end{array}$$

or alternatively, if we normalize with the known zero-rate shear value, $\eta_{0}=\frac{1}{60}\left(1\;+\;\frac{2}{3}\varepsilon \right)G\lambda$ [33,35], the result can also be written as

(7) $$\begin{array}{ccc} \frac{\eta_{1}\left(\dot{\epsilon }\right)}{\eta_{0}} & = & 3+b+\frac{240}{35\left(1\;+\;\frac{2}{3}\varepsilon \right)}\left(1\;-\;\frac{2b}{3}-\;\frac{b^{2}}{3}\right)\left(\;\frac{3}{4}+\varepsilon \right)\Delta_{1}\mathrm{Wi}\\ & & +\frac{720}{245\left(1\;+\;\frac{2}{3}\varepsilon \right)}\left(1\;+\;\frac{b^{2}}{3}\right)\left[\varepsilon \left(\Delta_{2}+6\Delta_{3}\right)\left(1\;+\;\frac{b}{3}\right)+\frac{3}{4}\left(1\;+\;\frac{b^{2}}{3}\right)\left(\Delta_{2}-8\Delta_{3}\right)\right]{\mathrm{Wi}}^{2},\\ \frac{\eta_{2}\left(\dot{\epsilon }\right)}{\eta_{0}} & = & 2b-\frac{960b}{105\left(1\;+\;\frac{2}{3}\varepsilon \right)}\left(\;\frac{3}{4}+\varepsilon \right)\Delta_{1}\mathrm{Wi}\\ & & +\frac{480b}{245\left(1\;+\;\frac{2}{3}\varepsilon \right)}\left(1\;+\;\frac{b^{2}}{3}\right)\left[\left(\frac{3}{4}+\varepsilon \right)\Delta_{2}+6\left(\varepsilon -1\right)\Delta_{3}\right]{\mathrm{Wi}}^{2}. \end{array}$$

This way we see that the first and second zero-rate elongation viscosities $\lim_{\mathrm{Wi}\to 0}\eta_{1,2}\left(\dot{\epsilon }\right)$ are 3+b and 2b times, respectively, the zero-rate shear viscosity, $\eta_{0}$. The following abbreviations have been introduced for numerical prefactors appearing in (6) and (7)

(8) $$\Delta_{j}\equiv 24\sum_{\nu =1,\mathrm{odd}}^{\infty }\frac{1}{{\left(\pi \nu \right)}^{4}k_{j}\left(\nu \right)},\;\;\;\left(j=1,2,3\right)$$

with the kernels $k_{1}\left(\nu \right)=K_{2}$, $k_{2}\left(\nu \right)=K_{2}^{2}$, and $k_{3}\left(\nu \right)=K_{2}K_{4}$ that depend on both $\varepsilon^{\prime }$ and $\varepsilon_{0}^{\prime }$ via

(9) $$K_{j}\left(\nu \right)\equiv \left(1-\varepsilon^{\prime }\right){\left(\pi \nu \right)}^{2}+j\left(j+1\right)\varepsilon_{0}^{\prime }.\;\;\;\left(j=1,2,3\right)$$

Evaluating the $\Delta_{j}$’s we can obtain more explicit predictions for two limiting cases (i) and (ii): (i) When $\varepsilon^{\prime }=0$, implying $K_{j}\left(\nu \right)={\left(\pi \nu \right)}^{2}$, the $\Delta_{j}$ are readily evaluated, $\Delta_{1}=1/40$ and $\Delta_{2}=\Delta_{3}=17/6720$, and Equation (6) reduces to

(10) $$\begin{array}{ccc} \frac{\eta_{1}\left(\dot{\epsilon }\right)}{G\lambda } & = & \frac{3+b}{60}\left(1\;+\;\frac{2}{3}\varepsilon \right)+\frac{1}{350}\left(1\;-\;\frac{2b}{3}-\;\frac{b^{2}}{3}\right)\left(\;\frac{3}{4}+\varepsilon \right)\mathrm{Wi}\\ & & +\frac{17}{19600}\left(1\;+\;\frac{b^{2}}{3}\right)\left[\varepsilon \left(1\;+\;\frac{b}{3}\right)-\;\frac{3}{4}\left(1\;+\;\frac{b^{2}}{3}\right)\right]{\mathrm{Wi}}^{2},\\ \frac{\eta_{2}\left(\dot{\epsilon }\right)}{G\lambda } & = & \frac{b}{30}\left(1\;+\;\frac{2}{3}\varepsilon \right)-\frac{2b}{525}\left(\;\frac{3}{4}+\varepsilon \right)\mathrm{Wi}\\ & & +\frac{17b}{29400}\left(1\;+\;\frac{b^{2}}{3}\right)\left(\varepsilon -\frac{3}{4}\right){\mathrm{Wi}}^{2},\;\;\;\;\left(\varepsilon^{\prime }=0\right) \end{array}$$

up to order ${\mathrm{Wi}}^{3}$. These expressions (10) further include the Doi & Edwards results for ε=0. (ii) In the second limit, $\varepsilon^{\prime }=1$ with N=2, thus $\varepsilon_{0}^{\prime }=\varepsilon^{\prime }$, the chain reduces to a rigid dumbbell. For this case $K_{j}\left(\nu \right)=j\left(j+1\right)$ and all kernels $k_{j}$ are independent on ν, leading to $\Delta_{1}=1/24$, $\Delta_{2}=1/144$, and $\Delta_{3}=1/480$. We thus obtain from Equation (6)

(11) $$\begin{array}{ccc} \frac{\eta_{1}\left(\dot{\epsilon }\right)}{G_{\mathrm{rd}}\lambda_{\mathrm{rd}}} & = & \frac{3(3+b)}{5}\left(1+\frac{2}{3}\varepsilon \right)+\frac{1}{35}\left(1\;-\;\frac{2b}{3}-\;\frac{b^{2}}{3}\right)\left(\frac{3}{4}+\varepsilon \right){\mathrm{Wi}}_{\mathrm{rd}}\\ & & +\frac{1}{1050}\left(1\;+\;\frac{b}{3}\right)\left[\varepsilon \left(1\;+\;\frac{b}{3}\right)-\frac{3}{8}\left(1\;+\;\frac{b^{2}}{3}\right)\right]{\mathrm{Wi}}_{\mathrm{rd}}^{2},\\ \frac{\eta_{2}\left(\dot{\epsilon }\right)}{G_{\mathrm{rd}}\lambda_{\mathrm{rd}}} & = & \frac{6b}{5}\left(1+\frac{2}{3}\varepsilon \right)-\frac{4b}{105}\left(\frac{3}{4}+\varepsilon \right){\mathrm{Wi}}_{\mathrm{rd}}\\ & & +\frac{2b}{2275}\left(1\;+\;\frac{b^{2}}{3}\right)\left(\varepsilon -\frac{3}{8}\right){\mathrm{Wi}}_{\mathrm{rd}}^{2},\;\;\;\;\left(\varepsilon^{\prime }=\varepsilon_{0}^{\prime }=1\right) \end{array}$$

with ${\mathrm{Wi}}_{\mathrm{rd}}\equiv \dot{\epsilon }\lambda_{\mathrm{rd}}$, $G=6G_{\mathrm{rd}}$ and $\lambda =6\lambda_{\mathrm{rd}}$. To the best of our knowledge, this expansion for the rigid rod subjected to biaxial flows is provided here for the first time. Our result, Equation (11), also accounts for hydrodynamic interaction by identifying $\varepsilon =\lambda_{2}^{\left(2\right)}/\lambda_{2}^{\left(1\right)}$ [35].

### 4.2. Stationary Regime, Variable ε

If, instead of a constant link tension coefficient, we consider the variable link tension coefficient of the tumbling snake model given by $\varepsilon =\varepsilon_{0}S_{2}^{2}$ where $S_{2}^{2}=\frac{3}{2}\mathrm{tr}\left({\langle \mathrm{uu}\rangle }_{\mathrm{ani}}\cdot {\langle \mathrm{uu}\rangle }_{\mathrm{ani}}\right)$ is the uniaxial order parameter of the 2nd moment (here ${\langle \mathrm{uu}\rangle }_{\mathrm{ani}}={\langle \mathrm{uu}\rangle }^{\left(1\right)}-\frac{1}{3}I$) [34,38], then, up to second order in Wi we obtain

(12) $$\varepsilon \left(\dot{\epsilon }\right)=\varepsilon_{0}\frac{4(3+b^{2})}{75}{\left(\Gamma_{1}\mathrm{Wi}\right)}^{2},$$

where $\Gamma_{1}$ is a numerical coefficient

(13) $$\Gamma_{1}=12\sum_{\nu =1,\mathrm{odd}}^{\infty }\frac{1}{{\left(\pi \nu \right)}^{2}K_{2}},$$

that appeared in the Supplementary Equation (S1b) of Ref. [38]. The corresponding steady-state viscosities are given, up to $O({\mathrm{Wi}}^{3})$, by

(14) $$\begin{array}{ccc} \frac{\eta_{1}\left(\dot{\epsilon }\right)}{G\lambda } & = & \frac{3+b}{60}+\frac{4}{35}\left(1\;-\;\frac{2b}{3}-\;\frac{b^{2}}{3}\right)\;\mathrm{Wi}+\frac{9(\Delta_{2}-8\Delta_{3})}{245}\;{\left(\;1\;+\;\frac{b^{2}}{3}\right)}^{2}\;\;\;{\mathrm{Wi}}^{2}+\frac{2\left(3+b\right)\left(3+b^{2}\right)\varepsilon_{0}}{3375}{\left(\Gamma_{1}\mathrm{Wi}\right)}^{2},\\ \frac{\eta_{2}\left(\dot{\epsilon }\right)}{G\lambda } & = & \frac{b}{30}-\frac{12b}{105}\Delta_{1}\mathrm{Wi}+\frac{6b(\Delta_{2}-8\Delta_{3})}{245}\left(1\;+\;\frac{b^{2}}{3}\right){\mathrm{Wi}}^{2}+\frac{4b\left(3+b^{2}\right)\varepsilon_{0}}{3375}{\left(\Gamma_{1}\mathrm{Wi}\right)}^{2}. \end{array}$$

We refrain from writing down more explicit expressions for the special cases of (i) $\varepsilon^{\prime }=0$, using the $\Delta_{j}$’s mentioned above for this case, as well as $\Gamma_{1}=1/8$, and (ii) $\varepsilon^{\prime }=\varepsilon_{0}^{\prime }=1$, using $\Gamma_{1}=1/4$.

## 5. Transient Elongational Viscosities in the Linear Viscoelastic Regime

To study the transient viscosities after startup of flow we consider a time-dependent spherical harmonics expansion of the single-link distribution function around equilibrium, up to first order in Wi, to be able to obtain the linear viscoelastic analytical predictions; the procedure is described in the Supplementary Section A; the final expression for the expansion of the time-dependent single-link distribution function is given by Supplementary Equation (S4).

### 5.1. Transient Regime, Constant ε

Inserting this expansion into the stress tensor Expression (1), assuming a constant ε, we obtain analytical expressions for the time dependent viscosities, $\eta_{1}^{+}\left(t\right)$, and $\eta_{2}^{+}\left(t\right)$, which turn out to be 3+b and 2b times the time-dependent shear viscosity, that was first presented in [34],

(15) $$\begin{array}{c} \frac{\eta_{1}^{+}\left(t\right)}{G\lambda }=\left(3+b\right)\left[\frac{1}{60}\left(1\;+\;\frac{2}{3}\varepsilon \right)-\frac{1}{15}\Delta_{0}\left(t\right)\right],\\ \frac{\eta_{2}^{+}\left(t\right)}{G\lambda }=2b\left[\frac{1}{60}\left(1\;+\;\frac{2}{3}\varepsilon \right)-\frac{1}{15}\Delta_{0}\left(t\right)\right],\; \end{array}$$

where the following abbreviation has been introduced,

(16) $$\begin{array}{ccc} \Delta_{0}\left(t\right) & = & 24\sum_{\nu =1,\mathrm{odd}}^{\infty }\frac{\exp(-K_{2}\left(\nu \right)t/\lambda )}{{\left(\pi \nu \right)}^{4}}, \end{array}$$

with $K_{2}\left(\nu \right)$ from Equation (9). An important property of $\Delta_{0}\left(t\right)$ is its initial value, $\Delta_{0}\left(0\right)=1/4$. It decreases monotonically with time, initially with a slope of $-24[\left(2-\varepsilon^{\prime }\right)+\varepsilon_{0}^{\prime }]/\lambda$, and ultimately vanishes as t→∞. Taking the rigid dumbbell limit, $\varepsilon^{\prime }=1$ and N=2, the $\Delta_{0}\left(t\right)$ can be evaluated analytically and Equation (15) becomes

(17) $$\begin{array}{ccc} \frac{\eta_{1}^{+}\left(t\right)}{G_{\mathrm{rd}}\lambda_{\mathrm{rd}}} & = & \frac{3(3+b)}{5}\left[1\;+\;\frac{2}{3}\varepsilon -\exp\left(-\frac{t}{\lambda_{\mathrm{rd}}}\right)\right],\\ \frac{\eta_{2}^{+}\left(t\right)}{G_{\mathrm{rd}}\lambda_{\mathrm{rd}}} & = & \frac{6b}{5}\left[1\;+\;\frac{2}{3}\varepsilon -\exp\left(-\frac{t}{\lambda_{\mathrm{rd}}}\right)\right].\;\;\;\left(\varepsilon^{\prime }=\varepsilon_{0}^{\prime }=1\right) \end{array}$$

To the best of our knowledge, this is the first time this result for a rigid dumbbell is presented.

### 5.2. Transient Regime, Variable ε

If, instead of a constant link tension coefficient, we consider a variable link tension coefficient given as $\varepsilon =\varepsilon_{0}S_{2}^{2}$, the least order expansion with respect to Wi gives

(18) $$\varepsilon \left(\dot{\epsilon },t\right)=\varepsilon_{0}\frac{4(3+b^{2})}{75}{\mathrm{Wi}}^{2}{\left[\Gamma_{1}-\Gamma_{1}\left(t\right)\right]}^{2},$$

where the dimensionless $\Gamma_{1}\left(t\right)=12\sum_{\nu =1,\mathrm{odd}}^{\infty }\exp\left(-K_{2}t/\lambda \right)/{\left(\pi \nu \right)}^{2}K_{2}$ with $\Gamma_{1}\left(0\right)=\Gamma_{1}$, cf. Equation (13), is taken from the Supplementary Equation (S3b) of Ref. [38]. Putting this together, ε at small rates adn times increases quadratically with rate and time. The precise expression is

(19) $$\varepsilon \left(\dot{\epsilon },t\right)=\varepsilon_{0}\frac{3(3+b^{2})}{25}{\left(\dot{\epsilon }t\right)}^{2}.$$

As the variable link tension coefficient thus vanishes in the linear viscoelastic regime, the full time dependent planar elongation viscosities are given by Equation (15) evaluated at ε=0, i.e.,

(20) $$\begin{array}{ccc} \frac{\eta_{1}^{+}\left(t\right)}{G\lambda } & = & \left(3+b\right)\left(\frac{1}{60}-\frac{1}{15}\Delta_{0}\left(t\right)\right),\\ \frac{\eta_{2}^{+}\left(t\right)}{G\lambda } & = & 2b\left(\frac{1}{60}-\frac{1}{15}\Delta_{0}\left(t\right)\right). \end{array}$$

For times t≪λ, this expression reduces, with $\Delta_{0}\left(t\right)$ given by Equation (16), to

(21) $$\begin{array}{ccc} \frac{\eta_{1}^{+}\left(t\right)}{G\lambda } & = & \frac{3+b}{10}\left[2\left(1-\varepsilon^{\prime }\right)+\varepsilon_{0}^{\prime }\right]\frac{t}{\lambda },\\ \frac{\eta_{2}^{+}\left(t\right)}{G\lambda } & = & \frac{b}{5}\left[2\left(1-\varepsilon^{\prime }\right)+\varepsilon_{0}^{\prime }\right]\frac{t}{\lambda }.\;\;\;\left(t\ll \lambda \right) \end{array}$$

where use had been made of the already mentioned initial slope of $\Delta_{0}\left(t\right)$.

## 6. Brownian Dynamics Results for Planar Elongational Flow

Having derived analytical expressions for the various regimes, we now turn to the presentation of full rate- and time-dependent exact numerical results for the tumbling-snake model for the special case of planar elongational flow (b=1).

(22) $$\kappa =\dot{\epsilon }\left(\begin{array}{ccc} -1 & 0 & 0\\ 0 & 0 & 0\\ 0 & 0 & 1 \end{array}\right)$$

where polymers tend to align in *z*-direction to the expense of a compression in *x*-direction, while *y* is the neutral direction. All figures presented in this manuscript are generated using the variable link tension coefficient; predictions for the case of a constant ε are available in the Supplementary Section B. All types of biaxial flows discussed above can be studied using an identical procedure. Mixed flows pose no additional problem and amount to consider a more general κ or even a time-dependent κ(t) in Equation (3) of the Brownian dynamics algorithm (5). The analytical results will be used to test the simulation results, and to extend their validity to “infinitely” small rates and times, where simulation results tend to become more difficult to sample.

### 6.1. Steady-State Planar Elongation

**Figure 3:**
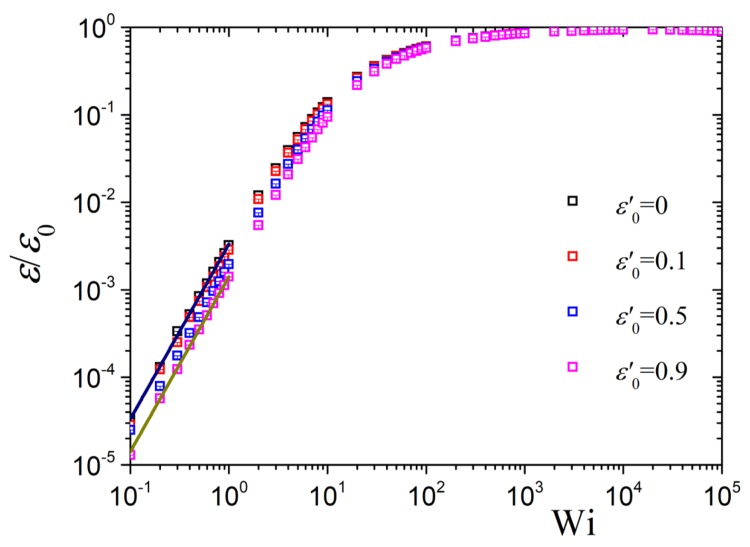
Predictions for the variable link tension coefficient, $\varepsilon /\varepsilon_{0}=S_{2}^{2}$ in planar elongational flow as a function of dimensionless elongation rate Wi for N=100 (Z≈33 entanglements) and various values of $\varepsilon_{0}^{\prime }$. The thick lines give the analytical predictions of Equation (23) when $\varepsilon_{0}^{\prime }=0$ (dark blue) and 0.9 (dark yellow). The dependency of $\Gamma_{1}$ on $\varepsilon_{0}^{\prime }$ determines their offsets and is relatively weak for any *N*. For N>2 the coefficient $\Gamma_{1}$ decreases monotonically with increasing $\varepsilon^{\prime }$, starting from $\Gamma_{1}=0.125$ at $\varepsilon^{\prime }=0$. For any N≥20 one has $\Gamma_{1}\approx 0.081$ at $\varepsilon^{\prime }=0.9$ (and $\Gamma_{1}\approx 0.078$ at $\varepsilon^{\prime }=1$).

The steady-state value of the variable link tension coefficient $\varepsilon =\varepsilon_{0}S_{2}^{2}$ as a function of dimensionless elongation rate Wi for polymerization degree N=100 (Z≈33 entanglements) and various values of $\varepsilon_{0}^{\prime }=\varepsilon^{\prime }{\left(N-1\right)}^{2}$ is shown in Figure 3. At small elongation rates, as expected from Equation (12) evaluated at b=1,

(23) $$\varepsilon \left(\dot{\epsilon }\right)=\varepsilon_{0}\frac{16}{75}{\left(\Gamma_{1}\mathrm{Wi}\right)}^{2},\;\;\;\left(b=1\right)$$

ε increases quadratically with the elongation rate, whereas at large Wi, and irrespective of the value of $\varepsilon_{0}^{\prime }$, then $\varepsilon \to \varepsilon_{0}$. This is also expected since for a fully aligned sample the order parameter approaches unity, $S_{2}\to 1$. Thus, the model predictions for the case of a variable ε become identical to the ones for a constant ε at large rates, Wi≫1.

**Figure 4:**
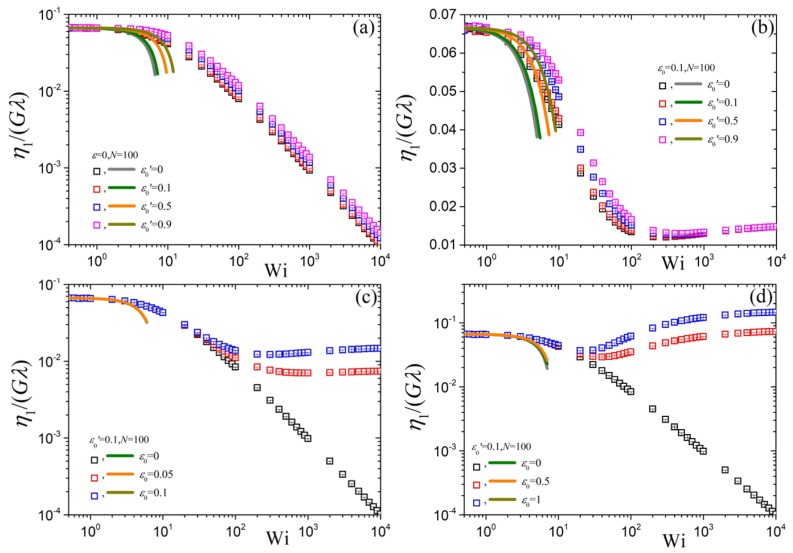
Predictions for $\eta_{1}\left(\dot{\epsilon }\right)/G\lambda$ for the tumbling snake model with variable ε as a function of dimensionless elongation rate Wi for N=100 and various values of the parameters $\varepsilon_{0}$ and $\varepsilon_{0}^{\prime }$. (**a**) $\varepsilon_{0}=0$, various $\varepsilon_{0}^{\prime }$, (**b**) $\varepsilon_{0}=0.1$, various $\varepsilon_{0}^{\prime }$, (**c**,**d**) $\varepsilon_{0}^{\prime }=0.1$, various $\varepsilon_{0}$. The solid lines mark the predictions of the small rate expansion, Equation (24), or Equation (25) for the case of $\varepsilon_{0}^{\prime }=0$. At large rates the viscosity approaches the value given by Equation (26).

The reduced steady-state first viscosity, $\eta_{1}\left(\dot{\epsilon }\right)$, as a function of the dimensionless elongation rate is presented in Figure 4. All solid lines in this figure are determined by Equation (14) evaluated at b=1, i.e.,

(24) $$\begin{array}{ccc} \frac{\eta_{1}\left(\dot{\epsilon }\right)}{G\lambda } & = & \frac{1}{15}+\frac{16}{245}\left(\Delta_{2}-8\Delta_{3}\right){\mathrm{Wi}}^{2}+\frac{32\varepsilon_{0}}{3375}{\left(\Gamma_{1}\mathrm{Wi}\right)}^{2},\\ \frac{\eta_{2}\left(\dot{\epsilon }\right)}{G\lambda } & = & \frac{1}{30}-\frac{12}{105}\Delta_{1}\mathrm{Wi}+\frac{8}{245}\left(\Delta_{2}-8\Delta_{3}\right){\mathrm{Wi}}^{2}+\frac{16\varepsilon_{0}}{3375}{\left(\Gamma_{1}\mathrm{Wi}\right)}^{2}.\;\left(b=1\right) \end{array}$$

with numerical prefactors $\Delta_{j}^{\prime }$s given by Equation (8). The corresponding predictions of the simplified tumbling-snake model ($\varepsilon^{\prime }=0$) read,

(25) $$\begin{array}{ccc} \frac{\eta_{1}\left(\dot{\epsilon }\right)}{G\lambda } & = & \frac{1}{15}-\frac{17}{14700}{\mathrm{Wi}}^{2}+\frac{\varepsilon_{0}}{6750}{\mathrm{Wi}}^{2}\\ \frac{\eta_{2}\left(\dot{\epsilon }\right)}{G\lambda } & = & \frac{1}{30}-\frac{1}{350}\mathrm{Wi}-\frac{17}{29400}{\mathrm{Wi}}^{2}+\frac{\varepsilon_{0}}{13500}{\mathrm{Wi}}^{2},\;\;\left(b=1,\varepsilon^{\prime }=0\right) \end{array}$$

where we have used the numerical values for $\Delta_{2}$ and $\Delta_{3}$ quoted after Equation (6), and $\Gamma_{1}=1/8$ for $\varepsilon^{\prime }=0$.

Figure 4a shows the variation of $\eta_{1}\left(\dot{\epsilon }\right)$ upon changing $\varepsilon_{0}^{\prime }$ (or $\varepsilon^{\prime }$) while keeping N=100 and ε=0 fixed. The prediction at small elongation rates is independent of the value of $\varepsilon_{0}^{\prime }$ and approaches the value $4\eta_{0}$. However, as Wi increases the first planar viscosity shear-thins after about Wi≈1. This is in disaccord with the predictions for the uniaxial elongation viscosity of the tumbling-snake model, $\eta_{E}$, which seems to be passing from a maximum when $\varepsilon_{0}^{\prime }>0$ (see inset of Figure 2a of Ref. [39]). However, both share the same power-law behavior at large elongation rates which is always equal to −1 irrespective of the value of $\varepsilon_{0}^{\prime }$, as for the Doi & Edwards model [18].

In Figure 4b we show the same variation as in Figure 4a but now with $\varepsilon_{0}=0.1$. Again, $\eta_{1}\left(\dot{\epsilon }\right)$ follows Equation (24), or Equation (25) for the case of $\varepsilon_{0}^{\prime }=0$, at small elongation rates and, irrespective of the value of $\varepsilon_{0}^{\prime }$, reaches monotonically the same value at large elongation rates. This value is simply equal to

(26) $$\frac{\eta_{1}\left(\infty \right)}{G\lambda }\equiv \lim_{\mathrm{Wi}\to \infty }\frac{\eta_{1}\left(\dot{\epsilon }\right)}{G\lambda }=\frac{\varepsilon_{0}}{6}\;\Rightarrow \;\frac{\eta_{1}\left(\infty \right)}{4\eta_{0}}=\frac{5\varepsilon_{0}}{2}\;\;\left(b<3\right)$$

where $\eta_{0}=G\lambda /60$ is the zero rate shear viscosity for the case of variable ϵ, that vanishes at vanishing rate. The reduced first planar viscosity thus drops (or rises) from a value of 1/15 at small rates to $\varepsilon_{0}/6$ at infinitely large ones. For the scenario $\varepsilon_{0}=0.1$ shown in Figure 4b, the reduced first planar viscosity reduces to $\eta_{1}\left(\infty \right)/G\lambda =$ 1/60. Equation (26) can be readily derived by noting that as Wi→∞ then $\lim_{\mathrm{Wi}\to \infty }\;\varepsilon =\varepsilon_{0}$ as well as

(27) $$\begin{array}{ccc} \lim_{\mathrm{Wi}\to \infty }\;{\langle u_{z}^{2}-u_{x}^{2}\rangle }^{\left(1\right)} & = & 1\\ \lim_{\mathrm{Wi}\to \infty }\;{\langle u_{z}^{2}-u_{x}^{2}\rangle }^{\left(2\right)} & = & 1/6,\;\;(b<3)\\ \lim_{\mathrm{Wi}\to \infty }\;B_{zz}-B_{xx}\; & = & \mathrm{Wi}/6. \end{array}$$

These expressions hold as long as $\kappa_{zz}$ is the largest diagonal component of κ, which is the case for any b<3. Using similar arguments, $\eta_{2}\left(\infty \right)=0$ for b<3. Equation (27) are analogous to the case of uniaxial elongation [39], apply independently of the value of $\varepsilon_{0}^{\prime }$, and originate from the dominance of the third term in the stress tensor expression, Equation (1), at large elongation rates leading to a leveling-off of the first viscosity at a value given by Equation (26). By further increasing the value of the parameter $\varepsilon_{0}$, for given $\varepsilon_{0}^{\prime }$ and *N*, the small elongation rate predictions are unaffected (Figure 4c,d). At large Wi the curves always reach the value of the reduced $\eta_{1}\left(\infty \right)$, Equation (26). When the value of $\varepsilon_{0}$ exceeds 2/5 then $\eta_{1}\left(\infty \right)>4\eta_{0}$ (Figure 4d). The corresponding predictions when the link tension coefficient is considered constant, but non-vanishing, are given in Supplementary Figure S1.

**Figure 5:**
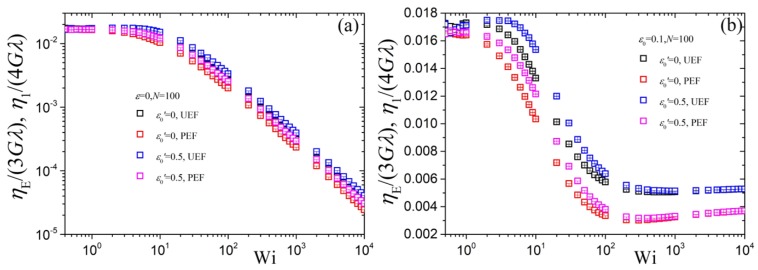
Comparison of the predictions for the first planar $\eta_{1}\left(\dot{\epsilon }\right)/4G\lambda$ (black, blue, denoted as PEF in the legends) and uniaxial $\eta_{E}\left(\dot{\epsilon }\right)/3G\lambda$ (red, pink, UEF) reduced viscosities as a function of dimensionless elongation rate Wi for N=100 and two values of the parameter $\varepsilon_{0}^{\prime }$. (**a**) $\varepsilon_{0}=0$, (**b**) $\varepsilon_{0}=0.1$.

From a visual comparison between planar $\eta_{1}\left(\dot{\epsilon }\right)$ and uniaxial $\eta_{E}\left(\dot{\epsilon }\right)$ (see Figure 2 of Ref. [39]) we note that their predictions are similar. To better quantify the similarities between $\eta_{1}$ and $\eta_{E}$ we compare the two in Figure 5 in a way so that they both have the same zero-rate prediction. We show the comparison upon changing $\varepsilon_{0}^{\prime }$ whilst keeping N=100 and ε=0 (Figure 5a) or $\varepsilon_{0}=0.1$ (Figure 5b) fixed. We find that $\eta_{E}\left(\dot{\epsilon }\right)/3G\lambda$ is slightly exceeding $\eta_{1}\left(\dot{\epsilon }\right)/4G\lambda$ in the intermediate flow regime, starting at about Wi≈3. Further, $\eta_{1}\left(\dot{\epsilon }\right)/4G\lambda$ for $\varepsilon_{0}^{\prime }=0.5$ is basically coinciding with $\eta_{E}\left(\dot{\epsilon }\right)/3G\lambda$ for $\varepsilon_{0}^{\prime }=0$. For the case $\varepsilon_{0}>0$ in Figure 5b we note that at large elongation rates $\eta_{E}\left(\infty \right)/3G\lambda$ reaches a constant value larger than the corresponding one for planar elongation, $\eta_{1}\left(\infty \right)/4G\lambda$, which stems from the way the two viscosities were made dimensionless; if both were made dimensionless with Gλ the corresponding value would be the same for both viscosities.

**Figure 6:**
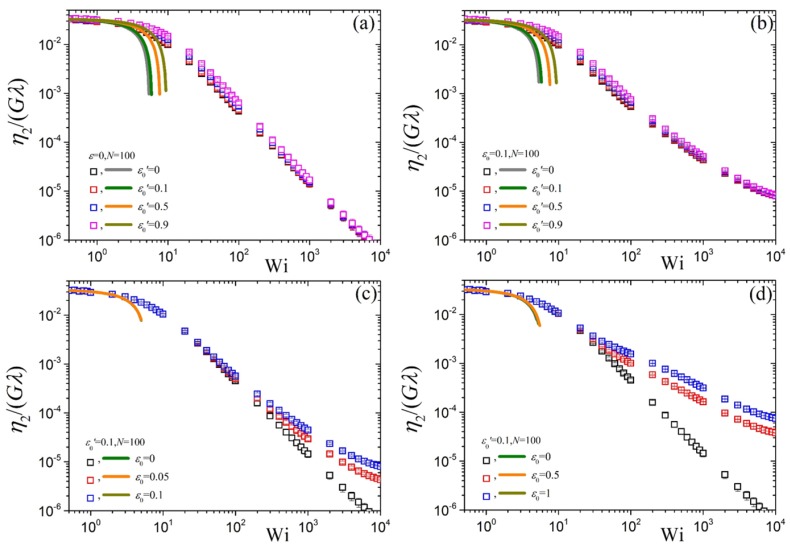
Predictions for $\eta_{2}\left(\dot{\epsilon }\right)/G\lambda$, analogous to Figure 4, as a function of dimensionless elongation rate Wi for N=100 and various values of the parameters $\varepsilon_{0}$ and $\varepsilon_{0}^{\prime }$. (**a**) $\varepsilon_{0}=0$, various $\varepsilon_{0}^{\prime }$, (**b**) $\varepsilon_{0}=0.1$, various $\varepsilon_{0}^{\prime }$, (**c**,**d**) $\varepsilon_{0}^{\prime }=0.1$, various $\varepsilon_{0}$. The solid lines mark the predictions of the small rate expansion, Equation (24), or Equation (25) for the case of $\varepsilon_{0}^{\prime }=0$.

The reduced steady-state second viscosity, $\eta_{2}\left(\dot{\epsilon }\right)$, as a function of the dimensionless elongation rate is presented in Figure 6. As for $\eta_{1}\left(\dot{\epsilon }\right)$, the prediction at small elongation rates is independent of the values of $\varepsilon_{0}^{\prime }$ and $\varepsilon_{0}$ in accord with Equation (24), or Equation (25) for the case of $\varepsilon_{0}^{\prime }=0$, and approaches the value $2\eta_{0}$. Also, like $\eta_{1}\left(\dot{\epsilon }\right)$, as Wi increases the second viscosity shear-thins after about Wi≈1. It can be noted that the predictions when $\varepsilon_{0}$ is kept fixed (panels a and b) are almost the same irrespective of the value of $\varepsilon_{0}^{\prime }$ and the power-law behavior at large elongation rates when ε=0 is equal to −3/2 as for the Doi & Edwards model. In panels Figure 6c,d we note that as the $\varepsilon_{0}$ increases under fixed $\varepsilon_{0}^{\prime }$, $\eta_{2}\left(\dot{\epsilon }\right)$ increases after about Wi≈20.

### 6.2. Transient Planar Elongation

**Figure 7:**
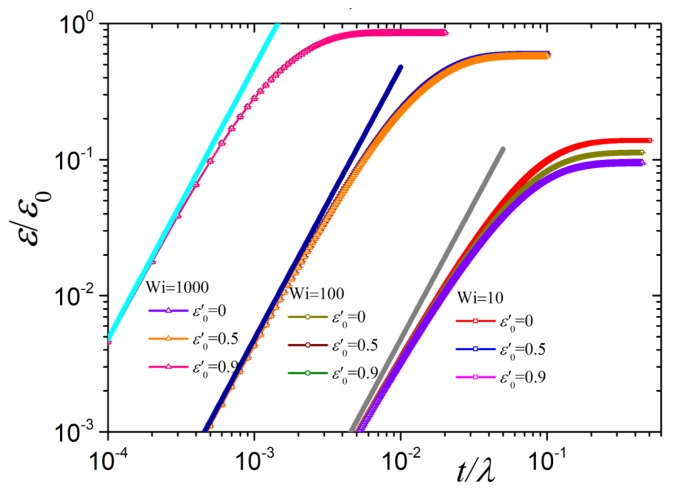
Predictions for the link tension coefficient, $\varepsilon \left(t\right)/\varepsilon_{0}$ as a function of dimensionless time for N=100 and various values of the parameter $\varepsilon_{0}^{\prime }$ and dimensionless elongation rate Wi. The thick straight lines give the prediction of $\frac{12}{25}{\left(\dot{\epsilon }t\right)}^{2}$ according to Equation (19) with b=1.

Next we inspect the transient link tension coefficient, $\varepsilon /\varepsilon_{0}$, as a function of dimensionless time t/λ for N=100 and various values of the parameter $\varepsilon_{0}^{\prime }$ and dimensionless elongation rates Wi (Figure 7). At early times, this coefficient follows $\frac{12}{25}{\left(\dot{\epsilon }t\right)}^{2}$ according to Equation (19) with b=1, irrespective of Wi and $\varepsilon_{0}^{\prime }$, whereas at larger times it monotonically approaches the steady-state values of the squared order parameter $S_{2}^{2}$. A similar behavior was also noted for the transient behavior of the variable link tension coefficient in uniaxial elongation [39].

**Figure 8:**
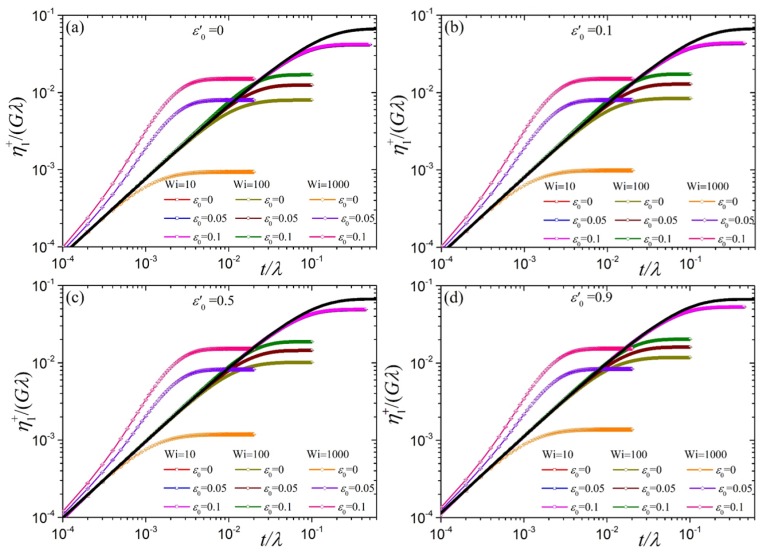
Predictions for $\eta_{1}^{+}\left(\dot{\epsilon },t\right)/G\lambda$ for the model with variable ε as a function of time for N=100 and various values of the parameter $\varepsilon_{0}$ and the dimensionless elongation rate Wi for (**a**) $\varepsilon_{0}^{\prime }=0$, (**b**) $\varepsilon_{0}^{\prime }=0.1$, (**c**) $\varepsilon_{0}^{\prime }=0.5$, and (**d**) $\varepsilon_{0}^{\prime }=0.9$. The solid black lines represent the linear viscoelastic results, Equation (28), their initial slopes are given by the right hand side of this equation with $\varepsilon^{\prime }=\varepsilon_{0}^{\prime }/{\left(N-1\right)}^{2}$. For the model with constant ε the transient viscosities do not vanish in the limit t/λ→0 (see Supplementary Section B).

In Figure 8 we show the transient first viscosity $\eta_{1}^{+}\left(\dot{\epsilon },t\right)$ as a function of the dimensionless time for various dimensionless elongation rates along with the linear viscoelastic prediction, that follows from Equation (20) with b=1,

(28) $$\begin{array}{ccc} \frac{\eta_{1}^{+}\left(t\right)}{G\lambda } & = & 4\left(\frac{1}{60}-\frac{1}{15}\Delta_{0}\left(t\right)\right)=\frac{2}{5}\left[2\left(1-\varepsilon^{\prime }\right)+\varepsilon_{0}^{\prime }\right]\frac{t}{\lambda }+O{\left(\frac{t}{\lambda }\right)}^{2},\\ \frac{\eta_{2}^{+}\left(t\right)}{G\lambda } & = & 2\left(\frac{1}{60}-\frac{1}{15}\Delta_{0}\left(t\right)\right)=\frac{1}{5}\left[2\left(1-\varepsilon^{\prime }\right)+\varepsilon_{0}^{\prime }\right]\frac{t}{\lambda }+O{\left(\frac{t}{\lambda }\right)}^{2}.\;\left(b=1\right) \end{array}$$

**Figure 9:**
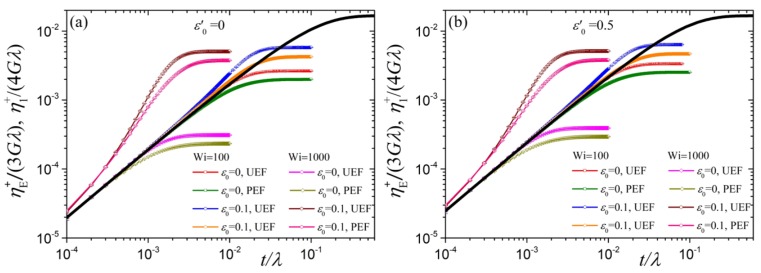
Comparison between the predictions for $\eta_{1}^{+}\left(\dot{\epsilon },t\right)/4G\lambda$ (denoted as PEF in the legends) and $\eta_{E}^{+}\left(\dot{\epsilon },t\right)/3G\lambda$ (UEF) as a function of dimensionless elongation rate Wi for N=100 for the model with variable link-tension coefficient ε. (**a**) $\varepsilon_{0}^{\prime }=0$, (**b**) $\varepsilon_{0}^{\prime }=0.5$.

The first term on the right hand side provides the full dependency on time, the second the initial slope. For all elongation rates we indeed notice from our Brownian dynamics results that as t→0 the transient first viscosity $\eta_{1}^{+}\left(\dot{\epsilon },t\right)/G\lambda$ increases with increasing $\varepsilon_{0}^{\prime }$, as suggested by the slope provided by Equation (28). As was the case for the shear viscosity [34,38] and the uniaxial elongation viscosity [39], by using the variable link tension coefficient $\varepsilon =\varepsilon_{0}S_{2}^{2}$ we have amended the problematic predictions of the original tumbling-snake model, in which a constant link tension coefficient is considered, according to which both limiting $\eta_{1}^{+}\left(t\right)/G\lambda$ and $\eta_{2}^{+}\left(t\right)/G\lambda$ approach a constant value, $\varepsilon_{0}/15$ and $\varepsilon_{0}/30$, respectively, irrespective of the value of the parameter $\varepsilon_{0}^{\prime }$ and thus the mode, reptation versus orientational diffusion. Additional Figures S3 and S4 are provided in Supplementary Section B. By further increasing the elongation rate (Wi=100) the transient first viscosity goes over the linear viscoelastic prediction only when $\varepsilon_{0}>0$, and reaches the steady-state value without reaching an overshoot, independently of the value of the parameters $\varepsilon_{0}^{\prime }>0$ and $\varepsilon_{0}$. We observed a similar trend in the case of uniaxial $\eta_{E}^{+}\left(\dot{\epsilon },t\right)$[39]. Similarly for larger elongation rate (Wi=1000) but now the curves depart much sooner and more intensely from the linear viscoelastic prediction, just like $\eta_{E}^{+}\left(\dot{\epsilon },t\right)$ does depart from $\eta_{E}^{+}\left(t\right)$. The non-appearance of an undershoot in shearfree flows is attributed to the absence of a rotational contribution to κ, and because the orientational diffusion of segments does therefore not lead to any deterministic rotation. On the other hand, under shear the tumbling behavior of polymer chains is not only due to the rotational component of κ, but triggered by the orientational diffusion term, present for $\varepsilon_{0}^{\prime }>0$. It produces undershoots at large shear rates [34,38]. Overall, the predictions for the transient first viscosity exhibit the same time-dependency with the uniaxial elongation viscosity except at large times as they approach their steady-state values (Figure 9).

**Figure 10:**
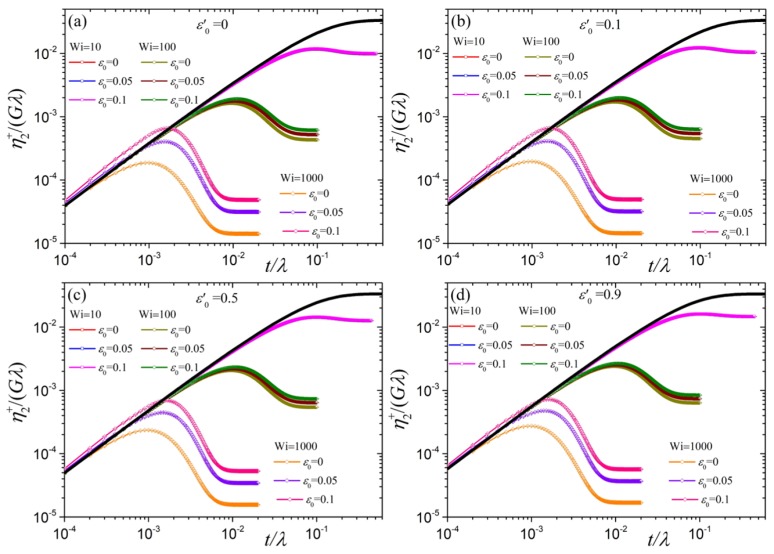
Predictions for $\eta_{2}^{+}\left(\dot{\epsilon },t\right)/G\lambda$, analogously to Figure 7 for $\eta_{1}^{+}\left(\dot{\epsilon },t\right)/G\lambda$, for the model with variable ε as a function of time for N=100 and various values of the parameter $\varepsilon_{0}$ and the dimensionless elongation rate Wi for (**a**) $\varepsilon_{0}^{\prime }=0$, (**b**) $\varepsilon_{0}^{\prime }=0.1$, (**c**) $\varepsilon_{0}^{\prime }=0.5$, and (**d**) $\varepsilon_{0}^{\prime }=0.9$. The solid black lines represent the linear viscoelastic results, Equation (28). For the model with constant ε the transient viscosities do not vanish in the limit t/λ→0 (see Supplementary Section B).

Finally, we investigate the transient second viscosity $\eta_{2}^{+}\left(\dot{\epsilon },t\right)$ as a function of the dimensionless time for various dimensionless elongation rates along with the linear viscoelastic predictions given by Equation (28) in Figure 10. Like $\eta_{1}^{+}\left(\dot{\epsilon },t\right)$, for all elongation rates and as t/λ→0 the transient second viscosity $\eta_{2}^{+}\left(\dot{\epsilon },t\right)$ increases with increasing $\varepsilon_{0}^{\prime }$, following Equation (28). Also, at small times and irrespective of the elongation rate and the values of the parameters $\varepsilon_{0}^{\prime }$ and $\varepsilon_{0}$, $\eta_{2}^{+}\left(\dot{\epsilon },t\right)$ follows the linear viscoelastic prediction, Equation (28). It implies that the ratio between the two viscosities is two, initially. The most important distinctions between the transient behavior of $\eta_{1}^{+}\left(\dot{\epsilon },t\right)$ and $\eta_{2}^{+}\left(\dot{\epsilon },t\right)$ are the appearance of an overshoot, independently of the values of the parameters $\varepsilon_{0}^{\prime }$ and $\varepsilon_{0}$, and the fact that for $\varepsilon_{0}>0$ and any $\varepsilon_{0}^{\prime }$ the curves are only slightly above the linear viscoelastic curve at small times. As for $\eta_{1}^{+}\left(\dot{\epsilon },t\right)$, an undershoot is not predicted, as expected.

Finally, it should be noted that, as was the case for shear flow and UE, model predictions for constant values of the parameters $\varepsilon_{0}^{\prime }$ and $\varepsilon_{0}$ but with a different number of Kuhn segments *N* are found to be identical, for both steady-state and transient quantities, for large values of *N* (N≥10), since the two viscosities were scaled with the modulus *G* and the relaxation time λ, both of which do depend on *N*; thus, this comparison is not shown.

## 7. Conclusions

In this work, we discussed the features of the tumbling-snake model for concentrated solutions and entangled polymer melts subjected to both steady-state and transient biaxial elongation, focussing on planar elongation as an application of the more general framework. The model employs a variable link tension coefficient, given by $\varepsilon =\varepsilon_{0}S_{2}^{2}$[34,38], which, as for shear flow and uniaxial elongation, has amended several shortcomings of a constant link tension coefficient originally suggested by Bird et al. [33,37]. In particular, the two planar transient elongation viscosities $\eta_{1,2}^{+}\left(\dot{\epsilon },t\right)$ do not approach a finite value as t→0 upon using the variable link tension coefficient. We have shown that the predictions of the first planar viscosity $\eta_{1}$ and the uniaxial elongation viscosity are similar with respect to their transient and stationary behaviors, in accord with available experimental data [7]. On the other hand, the steady-state second planar viscosity $\eta_{2}$ always thins irrespective of the value of the ultimate strength of the link-tension coefficient, $\varepsilon_{0}$. Overall the second viscosity seems to share many features with shear viscosity with respect to rate and time, except that $\eta_{2}$, unlike the shear viscosity [34,38], does not (and should not) exhibit an undershoot in the course of time after startup of flow.

As a concluding remark, the tumbling-snake model with variable link-tension coefficient has been shown to provide a very adequate description of the available rheological measurements of entangled polymer melts and concentrated polymer solutions when subjected to shear [34,35,38], uniaxial elongation [39], and planar elongation. The model is tractable analytically at small and large rates, that are unaccessible or difficult to access by numerical inspection, and for all intermediate rates the model is solved conveniently by simple Brownian dynamics. Introducing further refinements, such as contour length fluctuations (see e.g., [23,47,48] and references therein), convective constraint release [27,28,29], flow-induced alignment of chain ends [46,49], and chain stretch [25,50], the latter being significant in strong elongation flows, could further improve the tumbling-snake’s model capacity to quantitatively predict the rheological response of entangled polymer melts and concentrated polymer solutions. As for our works preceding the present study, no such refinements were attempted to present predictions of the tumbling-snake model for future reference. This includes our analytical results for biaxial elongation in the case of purely one-dimensional diffusion, $\varepsilon_{0}^{\prime }=0$, or the transient viscosities for a rigid dumbbell, as such results were apparently not available so far.

## Supplementary Materials

The following are available online at http://www.mdpi.com/2073-4360/10/3/329/s1, Section A: Methodology to obtain the real spherical harmonics expansion of the single-link distribution function, Section B: Results of the BD simulations when a constant link tension coefficient is employed (Figures S1–S4), Figure S1: Steady-state first planar viscosity, Figure S2: Steady-state second planar viscosity, Figure S3: Transient first planar viscosity, Figure S4: Transient second planar viscosity.
