# Supplementary material for: Tumbling-Snake Model for Polymeric Liquids Subjected to Biaxial Elongational Flows with a Focus on Planar Elongation
Source: Polymers (Basel). 2018 Mar 16;10(3):329. doi: 10.3390/polym10030329 (PMC6415193; doi:10.3390/polym10030329)
Supplement: Supplementary file 1 [file polymers-10-00329-s001.pdf]

SUPPLEMENTARY MATERIAL

Tumbling-snake model for polymeric liquids subjected to biaxial elongational flows with a focus on planar elongation

Pavlos S. Stephanou,[1*] and Martin Kröger[2*]

[1]Department of Mathematics and Statistics, University of Cyprus, PO Box 20537, 1678 Nicosia, Cyprus

[2]Department of Materials, Polymer Physics, ETH Zurich, CH-8093 Zurich, Switzerland

*Corresponding authors; electronic mail: stefanou.pavlos@ucy.ac.cy, mk@mat.ethz.ch

A. Methodology to obtain the real-valued spherical harmonics expansion of the single-link distribution function

Upon using for a general shearfree elongational flow

$$\begin{aligned}\mathrm{Wi}\, B_{nplm} &= -\int d\boldsymbol{u} y_{np}(\boldsymbol{u})\mathcal{L}\cdot\left[\boldsymbol{u}\times\tilde{\boldsymbol{\kappa}}\cdot\boldsymbol{u} y_{lm}(\boldsymbol{u})\right] \\ &= \mathrm{Wi}\int d\boldsymbol{u} y_{lm}(\boldsymbol{u})\left[b u_x u_y \mathcal{L}_z\left(y_{np}\right)-\tfrac{1}{2}(3+b)u_x u_z \mathcal{L}_y\left(y_{np}\right)+\tfrac{1}{2}(3-b)u_y u_z \mathcal{L}_x\left(y_{np}\right)\right],\end{aligned} \tag{A1}$$

in Eq. (C4) of Ref. [1] and performing the necessary integrations we obtain

$$\begin{aligned}&\frac{1}{2}\dot{b}_{00}^{(\mu)}(t)\delta_{n0}\delta_{p0}+\frac{1}{2}\dot{b}_{np}^{(\mu)}(t)= \\ &-\frac{1}{2}b_{00}^{(\mu),\mu\ \text{even}}(t)\left\{(1-\varepsilon')(\mu\pi)^2\delta_{n0}\delta_{p0}-\mathrm{Wi}B_{np00}\right\} \\ &-\frac{1}{2}\left[(1-\varepsilon')(\mu\pi)^2+\varepsilon_0' n(n+1)\right]b_{np}^{(\mu)}(t)+\frac{1}{2}\mathrm{Wi}\sum_{l=1}^{L}\sum_{m=-l}^{l}b_{lm}^{(\mu)}(t)B_{nplm} \\ &+\frac{3}{4\mu\pi^2}\left[1-(-1)^{\mu}\right]Wi\int d\boldsymbol{u} y_{np}(\boldsymbol{u})\left[-\frac{1}{2}(1+\beta)u_x^2-\frac{1}{2}(1-\beta)u_y^2+u_z^2\right]\end{aligned}. \tag{A2}$$

Note that already in the 2nd line of Eq. (A1) we have specified our treatment to the case of a general shearfree elongational flow, i.e. $\tilde{\boldsymbol{\kappa}}=\tilde{\boldsymbol{\kappa}}^T=\mathrm{diag}\left(-\tfrac{1}{2}(1+b),-\tfrac{1}{2}(1-b),1\right)\mathrm{Wi}$ where

Wi=$\dot{\varepsilon}\lambda$. Now, by taking cases separately we find that $b_{00}^{(\mu)}(t)=0,\forall\mu$ and $b_{np}^{(\mu)}(t)=0,\mu$ even,$\forall n,p$. For the rest,

$$\begin{aligned}\dot{b}_{np}^{(\mu)}(t)&=-\sum_{l=1}^{L}\sum_{m=-l}^{l}b_{lm}^{(\mu)}A_{nplm}+C_{np}\qquad(\text{odd }\mu)\\A_{nplm}&=K_l\delta_{nl}\delta_{mp}-\mathrm{Wi}\,B_{nplm}\\C_{np}&=\frac{6}{\mu\pi}\mathrm{Wi}\left\{\sqrt{\frac{1}{5\pi}}\delta_{n2}\delta_{p0}-\frac{b}{15\sqrt{\pi}}\delta_{n2}\delta_{p2}\right\}\end{aligned}\quad,\tag{A3}$$

where δ_{nl} is the Kronecker symbol (if n=l, $\delta_{nl}=1$, otherwise $\delta_{nl}=0$) and the shorthand notation $K_j\equiv(1-\varepsilon')(\nu\pi)^2+\varepsilon_0'j(j+1)$ is used. By following the methodology employed in Refs. [1,2] we obtain the following expression for the time-dependent single-link distribution function:

$$\begin{aligned}f(\sigma,\boldsymbol{u},t)=\frac{1}{4\pi}\Bigg[&1+4\mathrm{Wi}\sqrt{\frac{\pi}{5}}\{g_1(\sigma,0)-g_1(\sigma,t)\}y_{20}(\boldsymbol{u})\\&-4\mathrm{Wi}b\sqrt{\frac{\pi}{15}}\{g_1(\sigma,0)-g_1(\sigma,t)\}y_{22}(\boldsymbol{u})\Bigg]+\mathcal{O}(\mathrm{Wi}^2)\end{aligned}\quad,\tag{A4}$$

where

$$g_1(\sigma,t)=\sum_{\substack{\nu=1\\odd}}^{\infty}\frac{6\sin(\nu\pi\sigma)}{(\nu\pi)K_2}\exp(-K_2t).\tag{A5}$$

Note that for $t\to0$ the single-link distribution function becomes, as should, equal to the equilibrium one whereas, as $t\to\infty$ the single-link distribution function becomes identical to the one given below in Eq. (A6), up to first order terms. The single-link distribution function presented here generalizes the one presented in Ref. [3] which was only specified for uniaxial elongation (b=0). In the case of a stationary state, we are interested to have the solution up to third order in the dimensionless elongation rate. By solving the stationary limit of Eq. (A3) we obtain

$$f(\sigma,\boldsymbol{u})=\frac{1}{4\pi}\left\{1+4\mathrm{Wi}\sqrt{\frac{\pi}{5}}g_1(\sigma)\left(y_{20}(\boldsymbol{u})-\frac{b}{\sqrt{3}}y_{22}(\boldsymbol{u})\right)+4\mathrm{Wi}^2\sqrt{\frac{\pi}{5}}g_2(\sigma)\left[\frac{3}{7}\left(1-\frac{b^2}{3}\right)y_{20}(\boldsymbol{u})+\right.\right.$$

$$\left.\frac{6b}{7\sqrt{3}}y_{22}(\boldsymbol{u})\right]+4\mathrm{Wi}^2\sqrt{\frac{\pi}{5}}g_3(\sigma)\left[\frac{6\sqrt{5}}{7}\left(1+\frac{b^2}{18}\right)y_{40}(\boldsymbol{u})-\frac{10b}{7}y_{42}(\boldsymbol{u})+\frac{5b^2}{3\sqrt{7}}y_{44}(\boldsymbol{u})\right]$$

$$+4\mathrm{Wi}^3\sqrt{\frac{\pi}{5}}\frac{9}{49}\left(1+\frac{b^2}{3}\right)\left(y_{20}(\boldsymbol{u})-\frac{b}{\sqrt{3}}y_{22}(\boldsymbol{u})\right)g_4(\sigma)+4\mathrm{Wi}^3\sqrt{\frac{\pi}{5}}\left[\frac{18\sqrt{5}}{539}\left(1-\frac{4b^2}{9}\right)y_{40}+\frac{15b}{539}\left(1+\frac{b^2}{3}\right)y_{42}\right.$$

$$\left.-\frac{10b^2}{77\sqrt{7}}y_{44}\right]g_5(\sigma)+4\mathrm{Wi}^3\sqrt{\frac{\pi}{5}}\left[\frac{90}{11}\sqrt{\frac{5}{13}}\left(1+\frac{b^2}{6}\right)y_{60}-\frac{30b}{11}\sqrt{\frac{42}{13}}\left(1+\frac{b^2}{36}\right)y_{62}+\frac{5b^2}{11}\sqrt{\frac{35}{13}}y_{64}\right.$$

$$\left.\left.-5b^3\sqrt{\frac{35}{858}}y_{66}\right]g_6(\sigma)\right\}+\mathcal{O}\left(\mathrm{Wi}^4\right) \quad , \qquad \text{(A6)}$$

where

$$g_1(\sigma)=\sum_{\substack{\nu=1\\odd}}^{\infty}\frac{6}{(\nu\pi)K_2}\sin(\nu\pi\sigma),\ g_2(\sigma)=\sum_{\substack{\nu=1\\odd}}^{\infty}\frac{6}{(\nu\pi)K_2^2}\sin(\nu\pi\sigma),\ g_3(\sigma)=\sum_{\substack{\nu=1\\odd}}^{\infty}\frac{6}{(\nu\pi)K_2K_4}\sin(\nu\pi\sigma)$$

$$g_4(\sigma)=\sum_{\substack{\nu=1\\odd}}^{\infty}\frac{6(K_4-8K_2)}{(\nu\pi)K_2^3K_4}\sin(\nu\pi\sigma),\ g_5(\sigma)=\sum_{\substack{\nu=1\\odd}}^{\infty}\frac{6(10K_2+11K_4)}{(\nu\pi)K_2^2K_4^2}\sin(\nu\pi\sigma),$$

$$g_6(\sigma)=\sum_{\substack{\nu=1\\odd}}^{\infty}\frac{6}{(\nu\pi)K_2K_4K_6}\sin(\nu\pi\sigma) \quad . \qquad \text{(A7)}$$

Note that $g_1(\sigma)\equiv g_1(\sigma,0)$, where $g_1(\sigma,t)$ is the corresponding expression employed in Eq. (A5).

B. Results of the BD simulations when a constant link tension coefficient is employed

Fig. S1 shows the reduced steady-state first [Fig. S1] and second [Fig. S2] viscosities in the case of planar extension, made dimensionless with $G\lambda$, as a function of the dimensionless elongation rate, Wi. In the case of a constant link tension coefficient, the expansion of the two elongational viscosities in the case of planar elongation up to second order in Wi is obtained by setting b=1 in Eqs. (6) of the manuscript:

$$\frac{\eta_1(\dot{\varepsilon})}{G\lambda} = \frac{1}{15}\left(1+\frac{2}{3}\varepsilon\right) + \frac{64}{735}\left[\varepsilon\left(\Delta_2+6\Delta_3\right)+\frac{3}{4}\left(\Delta_2-8\Delta_3\right)\right]\mathrm{Wi}^2$$
$$\frac{\eta_2(\dot{\varepsilon})}{G\lambda} = \frac{1}{30}\left(1+\frac{2}{3}\varepsilon\right) - \frac{16}{105}\left(\varepsilon+\frac{3}{4}\right)\Delta_1\mathrm{Wi} + \frac{32}{735}\left[\varepsilon\left(\Delta_2+6\Delta_3\right)+\frac{3}{4}\left(\Delta_2-8\Delta_3\right)\right]\mathrm{Wi}^2, \quad \text{(B1)}$$

with numerical prefactors Δ_j given by Eq. (8) of the manuscript. When $\varepsilon' = 0$

$$\frac{\eta_1(\dot{\varepsilon})}{G\lambda} = \frac{1}{15}\left(1+\frac{2}{3}\varepsilon\right) + \frac{17}{11025}\left(\varepsilon-\frac{3}{4}\right)\mathrm{Wi}^2$$
$$\frac{\eta_2(\dot{\varepsilon})}{G\lambda} = \frac{1}{30}\left(1+\frac{2}{3}\varepsilon\right) - \frac{2}{525}\left(\varepsilon+\frac{3}{4}\right)\mathrm{Wi} + \frac{17}{22050}\left(\varepsilon-\frac{3}{4}\right)\mathrm{Wi}^2, \quad \text{(B2)}$$

These predictions are provided in Figs. S1 and S2. All curves, irrespective of the value of ε_0', reach the same value of the first viscosity at large elongation rates. This value is simply given by:

$$\frac{\eta_1(\infty)}{G\lambda} = \lim_{\mathrm{Wi}\to\infty}\frac{\eta_1(\dot{\varepsilon})}{G\lambda} = \frac{\varepsilon}{6} \Rightarrow \frac{\eta_1(\infty)}{\eta_0} = \frac{30\varepsilon}{3+2\varepsilon}, \quad \text{(B3)}$$

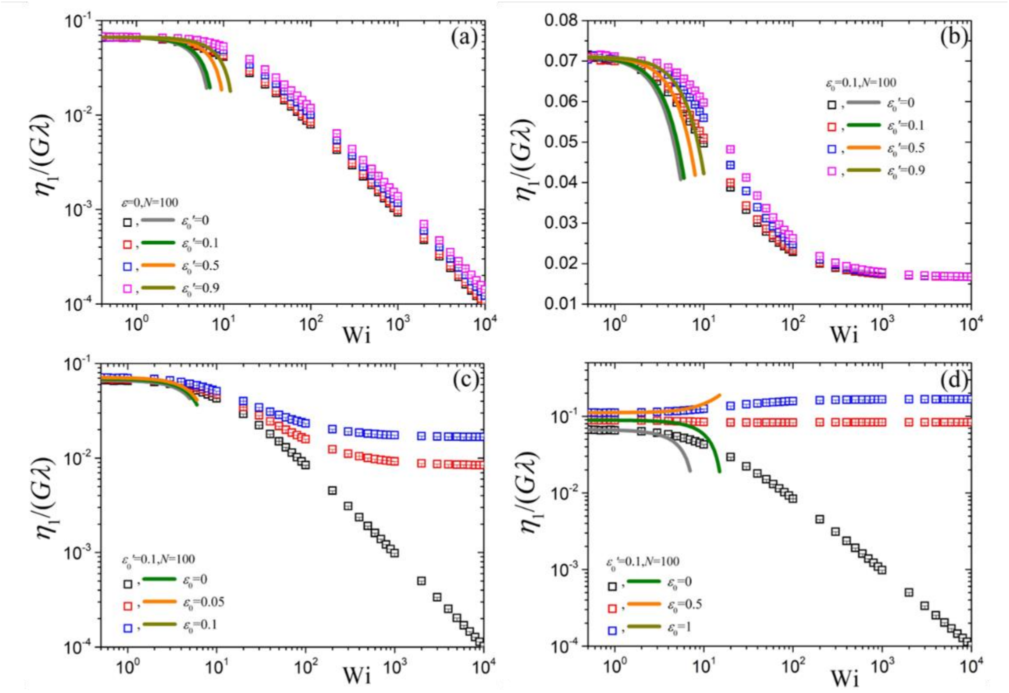

FIG. S1: $\eta_1/G\lambda$ as a function of Wi for various values of the parameters ε_0' and ε. Note that in part (b) and in all insets the vertical axis is in linear scale. The thick lines give the predictions of Eqs. (B1) or (B2) for the case of $\varepsilon_0' = 0$.

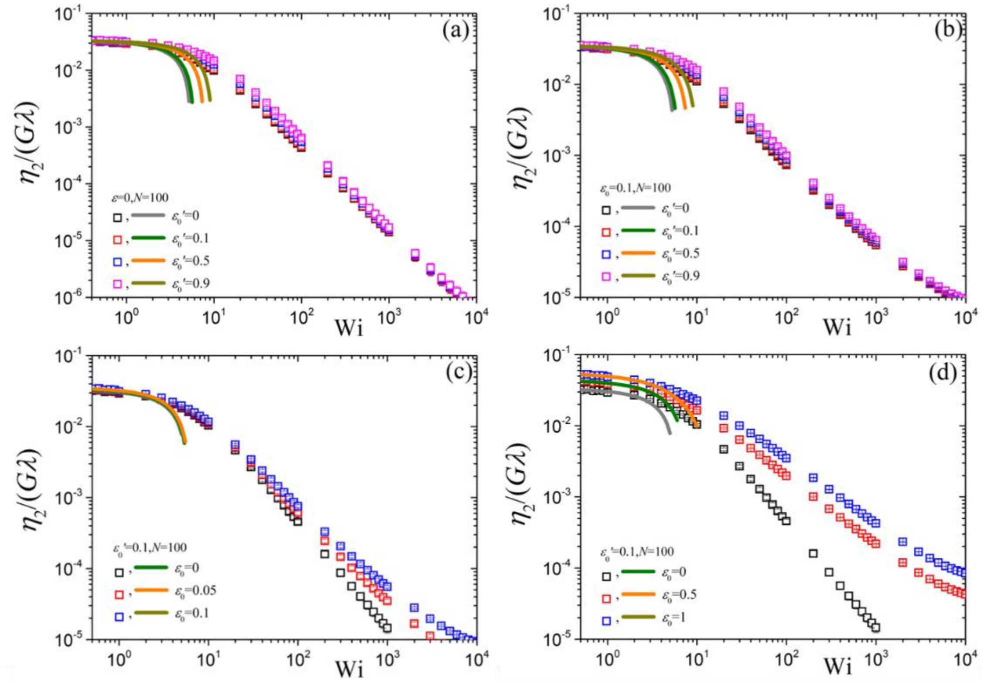

FIG. S2: Same as FIG. S1 but for $\eta_2/G\lambda$.

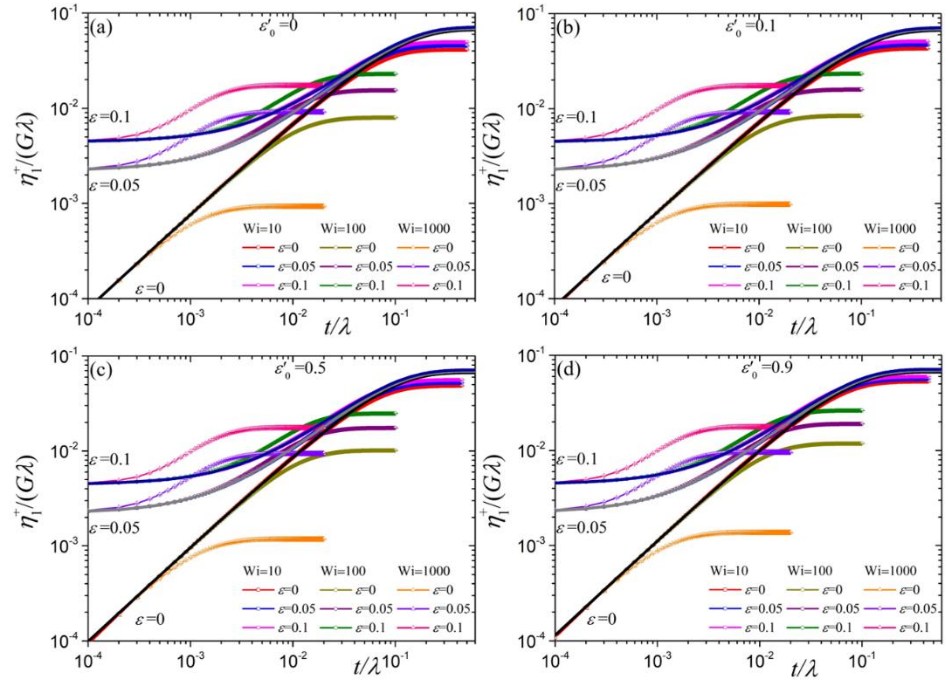

FIG. S3: Predictions for the transient $\eta_1^+/G\lambda$ as a function of time for N=100 and various values of the parameter ε and dimensionless elongation rate Wi for (a) ε_0'=0, (b) ε_0'= 0.1, c) ε_0'= 0.5, and (d) ε_0'= 0.9. The thick lines give the predictions of Eq. (14).

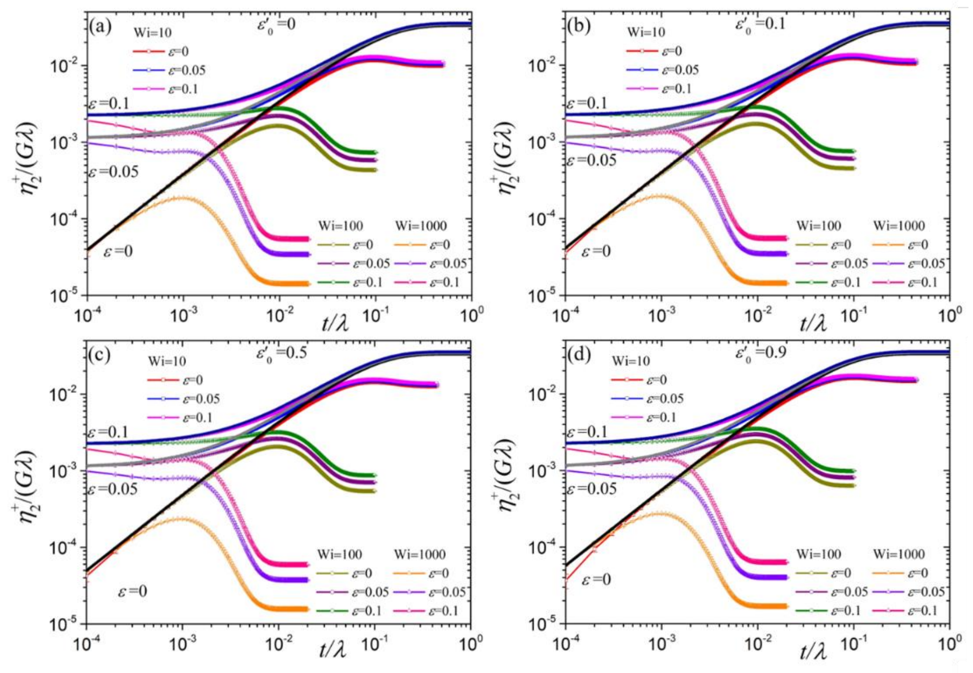

FIG. S4: Same as FIG. S3 but for $\eta_2^+/G\lambda$.

Note that Eq. (B3) differs from Eq. (26) only when the first viscosity is made dimensionless with the zero-rate viscosity which, when the link tension coefficient is taken as a constant, is given as

$$\eta_0 = \frac{G\lambda}{60}\left(1+\frac{2}{3}\varepsilon\right), \qquad \text{(B4)}$$

We next show the transient first (Fig. S3) and second (Fig. S4) viscosity as a function of the dimensionless time (t/λ) for various dimensionless elongation rates along with the linear viscoelastic prediction (see Eq. (15) with b=1).

References

1. P. S. Stephanou, T. Schweizer and M. Kröger, *J. Chem. Phys.* 146, 161101 (2017).
2. P. S. Stephanou and M. Kröger, *J. Chem. Phys*. 144, 124905 (2016)
3. P. S. Stephanou and M. Kröger, *J. Chem. Phys*. Under review (2018).
